# Impact of Federal, State, and Local Housing Policies on Disparities in Cardiovascular Disease in Black/African American Men and Women: From Policy to Pathways to Biology

**DOI:** 10.3389/fcvm.2022.756734

**Published:** 2022-04-18

**Authors:** Christopher Sistrunk, Nora Tolbert, Maria Dulfary Sanchez-Pino, Loretta Erhunmwunsee, Nikita Wright, Veronica Jones, Terry Hyslop, Gustavo Miranda-Carboni, Eric C. Dietze, Ernest Martinez, Sophia George, Augusto C. Ochoa, Robert A. Winn, Victoria L. Seewaldt

**Affiliations:** ^1^City of Hope Comprehensive Cancer Center, Duarte, CA, United States; ^2^Department of Cardiology, The University of North Carolina at Chapel Hill, Chapel Hill, NC, United States; ^3^Department of Interdisciplinary Oncology, Stanley S. Scott Cancer Center, Louisiana State University, Baton Rouge, LA, United States; ^4^Department of Biochemistry, Duke University, Durham, NC, United States; ^5^The University of Tennessee Health Science Center, Memphis, TN, United States; ^6^Department of Biostatistics and Bioinformatics, University of California, Riverside, Riverside, CA, United States; ^7^Sylvester Comprehensive Cancer Center, Miami, FL, United States; ^8^VCU Massey Cancer Center, Virginia Commonwealth University, Richmond, VA, United States

**Keywords:** redlining, African American, racial residential discrimination, structural racism, cardiovascular disease, obesity, PM_2.5_, GI bill

## Abstract

Racist and discriminatory federal, state, and local housing policies significantly contribute to disparities in cardiovascular disease incidence and mortality for individuals that self-identify as Black or African American. Here we highlight three key housing policies – “redlining,” zoning, and the construction of highways – which have wrought a powerful, sustained, and destructive impact on cardiovascular health in Black/African American communities. Redlining and highway construction policies have restricted access to quality health care, increased exposure to carcinogens such as PM_2.5_, and increased exposure to extreme heat. At the root of these policy decisions are longstanding, toxic societal factors including racism, segregation, and discrimination, which also serve to perpetuate racial inequities in cardiovascular health. Here, we review these societal and structural factors and then link them with biological processes such as telomere shortening, allostatic load, oxidative stress, and tissue inflammation. Lastly, we focus on the impact of inflammation on the immune system and the molecular mechanisms by which the inflamed immune microenvironment promotes the formation of atherosclerotic plaques. We propose that racial residential segregation and discrimination increases tissue inflammation and cytokine production, resulting in dysregulated immune signaling, which promotes plaque formation and cardiovascular disease. This framework has the power to link structural racism not only to cardiovascular disease, but also to cancer.

## Introduction

Structural racism is the “the normalization and legitimization of an array of dynamics – historical, cultural, institutional, and interpersonal – that routinely advantage non-Latino Whites while producing cumulative and chronic adverse outcomes for people of color” (1). Structural racism leads to unequal access to goods, services, and opportunities and underlies persistent health disparities in the United States (2). On November 10, 2020, the American Heart Association (AHA) issued a call to action. In this address, the AHA declared structural racism a key cause of premature death from cardiovascular disease (3) and called for change through strategic goals and increased focus on health equity (3).

The first scientific studies on health disparities showed that there were wide disparities in health (4, 5). These studies showed that Black/African Americans had earlier onset of disease, a more aggressive disease course, and worse survival compared to non-Latino Whites (6). These outcome differences persisted after adjusting for socioeconomic status (SES) (7). At every income and education level, Black/African Americans have a lower life expectancy than Whites (7). Recent scientific studies have identified many mechanisms by which structural, cultural, and individual racism can negatively impact health (5).

There is substantial evidence that racial discrimination is an important factor undermining the health of Black/African American men and women relative to Whites (2, 4, 5, 8, 9). For instance, the work of Lukachko et al. showed that structural racism increased the reported incidence of myocardial infarction in Black/African American men and women (9).

**FIGURE 1:**
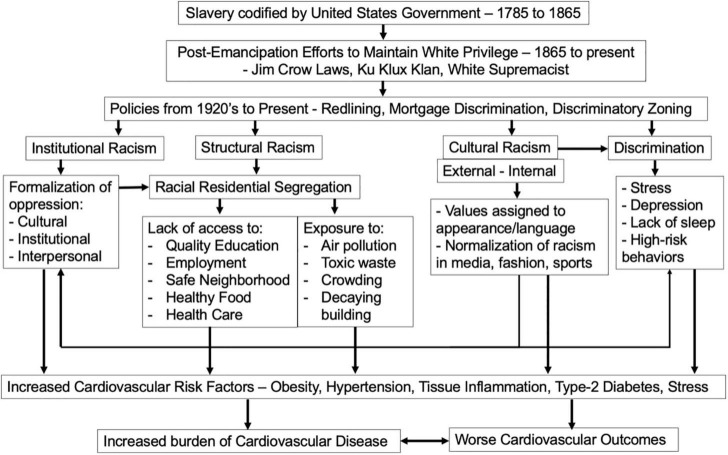
Linking slavery, racism, and discrimination on disparities in the incidence and outcomes cardiovascular disease in Black/African American individuals.

Although heart disease has declined over the past 50 years (10), the rate of decline has been significantly faster for non-Latino Whites than for Black/African Americans (10, 11). The Center for Disease Control (CDC) reported in 2017 that for Black/African Americans, the age-adjusted death rate for heart disease was 208 per 100,000, compared to 169 for non-Latino Whites (12). Many factors are responsible for these Black versus White cardiovascular disease disparities. In this review, we aim focus on how racist and discriminatory housing policies impact biological processes that increase cardiovascular disease (Figure 1).

## Historical Context – Legacy of Slavery and Jim Crow

Racism and discrimination have deep roots in the United States. The legacy of slavery and subsequent Jim Crow Laws have had a profound and lasting impact on the wealth, welfare, and health of Black/African American citizens. For a review, please see Churchwell et al. (3). In 1787, the newly formed United States, formally enslaved Black/African Americans and denied them all human rights. While not all states practiced slavery, the Fugitive Slave Act of 1859 allowed bounty hunters to kidnap former slaves seeking freedom in northern states and imposed stiff penalties on individuals aiding escaped slaves (13). The American Civil War brought emancipation to Black/African American slaves. However, the practices and codes of Southern states forced newly emancipated Black/African American individuals to work for little or no wages (3). In 1866, the Civil Rights Act gave Black/African American individuals limited property rights protection but not the right to vote or hold office. Meanwhile, these new legal protections fueled the paranoia of White supremacists, who enacted Jim Crow Laws and joined the Ku Klux Klan (14). These codes, laws, and discriminatory organizations form the basis of structural racism today and have long-acting consequences on housing, access, and health.

A landmark paper by Kramer et al. demonstrated the impact of slavery on heart disease mortality today (10). This study showed that Black/African Americans living in geographic regions of the United States with a historical legacy of slavery had worse survival from cardiovascular disease and fewer improvements in disease survival compared to geographic regions that didn’t practice slavery (10).

## Policies of the 1920’s to Today – Racism Manifested as National, State, and Local Policy Decisions That Impact Housing

**FIGURE 2:**
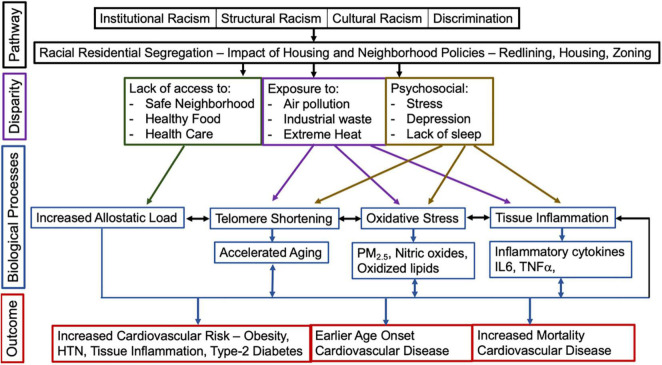
The impact of racist and discriminatory housing policies, disparities, biological processes on the risk for, the age of onset, and mortality experienced by Black/African American individuals from cardiovascular disease.

Racism is a social system organized by a dominant racial group, who make decisions based on a flawed ideology of inferiority (5, 15, 16). Through this faulty logic, the dominant majority acts to categorize and rank individuals and use its power to unequally allocate resources and opportunities to the group they define as inferior (5). Race is a social category, based on nationality, ethnicity, or physical characteristics (15). Racism affects individuals through multiple policy levels – national, state, and local (Figure 2) (5). Here we review housing policies that have had some of the most profound and sustained impact on cardiovascular health for Black/African American individuals living in the United States.

### Redlining of Black/African American Neighborhoods

The term “redlining” originally referred to a line in red ink that lenders placed on city maps around neighborhoods that were considered high-risk for mortgage default. “High-risk” neighborhoods were inevitably Black/African American or Latino/Hispanic. Today the term redlining refers to the systematic denial of services to residents of communities based on race and/or ethnicity. While refusals of home or business loans and insurance are the best-known examples of redlining, other services are subject to redlining including health care, grocery stores, and parks.

Redlining in the United States first took shape in the 1920’s (17–20). Maps outlined neighborhoods in four colors: green (Type A) were considered “most desirable” for lending, blue (Type B) were “still desirable,” yellow (Type C) were “declining,” and the “most-risky” neighborhoods were outlined in red (Type D). Type D, or “redlined,” areas were most frequently Black/African American neighborhoods (17–20). In order to receive FHA loan insurance (21, 22), lenders had to preferentially make loans for homes that resided in Type A or Type B neighborhoods and deny loans for home in Type D neighborhoods. The impact of this policy was devastating. Between 1945 and 1959, less than 2% of federally insured loans were granted to Black/African Americans (23–25).

Redlining resulted in the isolation and decay of Black/African American neighborhoods, particularly in inner cities (26). Neighborhoods deteriorated because families could not obtain loans to purchase houses. Families left for the suburbs. Since schools are financed by property taxes, the schools also declined in parallel with the neighborhoods (26). Unjust federal lending laws of the past also created unequal access to groceries and safe places to exercise, since grocery stores and parks were less likely to be located in redlined neighborhoods (27, 28). Despite anti-redlining laws, home loan applications continue to be denied because of race or ethnicity.

### Highways – “White Man’s Roads Through Black Men’s Homes”

In 1956, the Eisenhower Administration spearheaded the passage of the Federal-Aid Highway Act (29–31), the purpose of which was to build our nation’s interstate highway system. The interstates were primarily intended to bolster national defense but also ushered in a new era of automobiles and mobility. While cars reduced reliance on public transportation – and its linked discriminatory policies, particularly in the South – the building of our nation’s highways resulted in the destruction of many thriving Black/African American neighborhoods (29).

Under the pretense of “slum removal” and “urban renovation,” national and local planners routed highways through communities of color. Vibrant Black/African American neighborhoods were destroyed throughout the United States in cities such as Charlotte, Flint, Pittsburgh, and Los Angeles (29). In some places, entire Black/African American communities were destroyed. In other places, highways served to separate Black/African American and non-Latino White neighborhoods.

Many times, these urban planning decisions were made at the request of White residents who wanted to maintain segregation. The impact was financially devastating and persists to this day (29). The nation’s highways, while built in the 1950’s, remain an enduring example of structural racism.

### Zoning

In 1917, the Supreme Court overturned Louisville, Kentucky’s racial zoning ordinances in a case called *Buchanan v. Warley* (26). The Supreme Court ruled that racial zoning ordinances “interfered with the right of a property owner to sell to whomever he pleased” (26).

Yet, many places found ways to circumvent this ruling (26). States could follow the *Buchanan* decision by using “designation use” zoning – for instance, residential versus industrial zoning – to perpetuate racial segregation. Black/African American neighborhoods were zoned as “industrial,” meaning their homes could be divided into apartments, while non-Latino White neighborhoods were zoned as “single family residence,” meaning these homes could not be legally subdivided (26). Industrial zoning designation also permitted the building of factories and power plants within Black/African American neighborhoods and markedly increased exposure of Black/African Americans to carcinogens, industrial waste, soot, and air pollution (26). Zoning ordinances, independent of redlining, greatly contributed to the destruction of Black/African American neighborhoods and have played an important role in promoting cardiovascular disease, as well as cancer, due to increased exposure to a type of air pollution called PM_2.5_ (32).

### Impact of Neighborhood and Housing Policy Decisions on Cardiovascular Risk Factors

National, state, and local policies including redlining, highway construction, and zoning have had a significant and long-lasting effect on the cardiovascular health of Black/African American individuals (Figures 1, 2). Redlining has a profound impact on the ability of Black/African Americans to live in safe neighborhoods with access to grocery stores and high-quality health care. Lack of spaces to exercise makes it difficult to prevent obesity, hypertension, and cardiovascular disease. Lack of grocery stores increases reliance on fast food and convenience stores, which in turn increases the risk of obesity, hypertension, and type-2 diabetes. Lack of access to primary care physicians means that chronic illnesses go untreated or undetected, leading to more emergency room visits and death (3, 33). A study of census tracts in New York City in 2020 showed that historically redlined neighborhoods have higher rates of obesity and lower rates of health insurance (33, 34). A study by Mujahid et al. recently investigated, in the Multi-Ethnic Study of Atherosclerosis, the link between historic redlining and cardiovascular health (35). This study found evidence that living in historically redlined neighborhoods was associated with poor cardiovascular health, but only in Black/African American individuals (35). This study underscores the importance of further mechanistic work, including the impact of early life exposures on cardiovascular health.

Compounding these structural inequities is climate change. Extreme heat conditions have now become the norm and further threaten cardiovascular health for residents of formerly redlined neighborhoods (36). Throughout the United States, these neighborhoods experience significantly higher temperatures than neighborhoods that were previously designated “most desirable” for real estate loans (36). Temperature differentials experienced by formerly redlined neighborhoods correlate with a prevalence of impervious surface area and a lack of trees, water, and greenspace. High temperatures also worsen the impact of air pollution on health (36). As global warming continues to worsen, this only deepens the argument for the importance of community investment, greenspace, and parks in formerly redlined neighborhoods.

Highway placement also is an important driver of health. As highways have become increasingly congested, Black/African American neighborhoods are exposed to ever higher levels of harmful air pollution, including PM_2.5_ (fine particulate matter <2.5 microns in diameter) (32). Exposure to PM_2.5_ directly increases the risk of cardiovascular disease, type-2 diabetes, cancer, and death (32). Current evidence shows that PM_2.5_ initiates inflammation and oxidative stress, which results in the release of inflammatory cytokines and oxidized lipids. The inflammation caused by PM_2.5_ is thought to synergize with obesity-induced tissue inflammation, worsening insulin resistance and promoting cardiovascular disease.

These inequities persist and continue to impair quality of life and health. In a 2021 study of all United States metropolitan areas in 1980 and 2010, Firebaugh and Acciai recently demonstrated that for Black/African American individuals disparities in neighborhood poverty decreased faster than racial residential segregation (37). This highly impactful study showed that while the income gap between White and Black/African American individuals has declined, this decline has not translated to an equivalent decline in racial residential segregation. Some of this residential change was due to a sharp increase in low-income non-Latino Whites living in low-income neighborhoods (38). As this article states, “the end of black segregation is not at hand.”

## Pathways: Impact of Racism and Discrimination on Cardiovascular Health

The impact and persistence of racial disparities in health should be framed in the context of relatively stable racialized social structures that determine disease risk factors (e.g., environmental exposure), opportunity (e.g., wealth, education), and resources that drive health (e.g., access to care, health insurance). In 1997, Williams argued that upstream social factors and downstream socioeconomic effects were the fundamental causes of racial health disparities (15). The past 20 years have seen a systematic identification of these upstream factors that perpetuate racial inequities in health, including institutional and structural racism, cultural racism, and discrimination (5, 15). Together, these factors all impact cardiovascular health.

### Institutional and Structural Racism vs. Cardiovascular Health

Structural racism refers to racism that is embedded in: (1) federal, state, and local laws, (2) practices of institutions, and (3) attitudes and practices of societies, and that provides advantages to groups self-identified as superior and oppresses groups identified as inferior (5, 39). Structural racism is sometimes used interchangeably with institutional racism; however, the latter is defined more narrowly as the policies and practices within a society’s institutions that allow advantaged groups to succeed while disadvantage groups fall behind. The criminal justice system is a good example of institutional racism, whereas redlining is an example of structural racism (39–44). There are many forms of institutional and structural racism that impact health (45). Here, we highlight the impact of institutional and structural racism on health and increased disparities in cardiovascular health.

Above, we highlighted many of the national, state, and local policies that led to racial residential segregation: (1) redlining, (2) mortgage discrimination, and (3) discriminatory zoning, which increase exposure to environmental pollutants and excess heat and reduce access to healthy food, health care, and safe places to exercise outdoors. Racial residential segregation also results in segregated schools with lower per-student spending, fewer high-quality teachers, and fewer opportunities to access higher education and high-paying professional jobs (46). The resultant poverty compounds the lack of access to health care, particularly for managing type-2 diabetes and cardiovascular disease, by presenting challenges with transportation and paid time off work (9, 44, 47–49).

There are significant racial disparities in cardiovascular risk factors, incidence, and mortality between White and Black/African American men and women of all ages (5, 9, 50). Black/African Americans experience cardiovascular disease at a significantly earlier age than non-Hispanic Whites (3), due to the higher prevalence of cardiovascular risk factors, including hypertension, type 2 diabetes, and obesity (33). A review of 50 empirical studies found that racial residential segregation was associated with poorer health overall (44, 51–54).

A key study by Lukachko et al. tested for the association between cardiovascular events and four domains of structural racism: (1) political participation, (2) educational attainment, (3) employment and job status, and (4) judicial treatment (9). These studies showed that the incidence of self-reported myocardial infarction events was significantly higher in Black/African Americans living in states with high levels of structural racism than those living in low structural racism states. Conversely, White individuals living in high structural racism states had either lower or no difference in self-reported myocardial infarction, compared to Whites living in low structural racism states (11). These results raise the important and disturbing possibility that structural racism not only harms individuals who are identified by individuals in power as “inferior” but also may benefit the individuals in power who impose stigma and discrimination.

Over the past two decades, incidence of cardiovascular disease has decreased in all Americans. However, the rate of decrease is significantly less in Black/African Americans than non-Hispanic Whites. Data from the Atherosclerosis Risk in Communities Studies showed that the decline in cardiovascular disease among Black/African American men was half (-3.2%/y) that of non-Hispanic White men (-6.5%/y) (55). This lack of decline is thought to be linked overall with structural racism and specifically racial residential segregation (3, 33). The detrimental impact of racial residential segregation on cardiovascular risk for Black/African American adults is supported by studies showing that when White and Black/African Americans live in the same neighborhood, cardiovascular risk normalizes (56, 57). A recent study of racial residential segregation found that young Black/African American men (ages 24–34) could achieve equal cardiovascular health (as defined by the American Heart Association) as Whites, but only when the proportion of Whites living in the same neighborhood was 55% or greater (OR = 0.67, 95% CI = 0.49, 0.92). Taken together, these studies highlight the powerful impact of structural racism, specifically racial residential segregation, on cardiovascular health (54).

### Cultural Racism

Cultural racism has two components: (1) the assignment by individuals in power of inferior status of the language, symbols, physical features, values/religion, and language of a group they identify as “inferior,” and (2) the adoption of these views by society as a whole (5, 58, 59). These views can be external or internalized, and many avenues in society can re-enforce and “normalize” cultural racism, including entertainment, fashion, and the media (5, 59–62). These cultural forms of racism can act to marginalize the opinions of individuals who are targeted and justify the continuation of oppression by individuals in power. When internalized, cultural racism can have severe consequences, including discrimination in employment, poor quality health care, and lack of opportunity.

Williams et al. identified three pathways in which cultural racism can impact health: (1) societal policies, (2) individual-level unconscious bias, and (3) stereotyping (5). First, cultural racism can drive policies that create and reinforce disparities in housing, education, and health care. Past examples of policies emanating from cultural racism include redlining and unequal distribution of benefits from the GI bill (5, 59, 63). Second, cultural racism can also lead to individual unconscious bias in health care and worsen outcomes for patients of color (5). Black/African Americans are less likely to receive referrals to specialists, particularly for cardiovascular care, and implicit bias among physicians leads to poor communication, lack of identification of disease symptoms, and missed diagnoses (5, 64–67). Despite the recent focus on cultural racism and implicit bias (68), many physicians deny the existence and impact of cultural racism on the delivery of health care and health care outcomes (69).

### Discrimination and Self-Reported Discrimination

Discrimination can be either an action or an outcome (5). Discrimination as an action is the unequal treatment due to race and/or ethnicity, and this discrimination can be unconscious or deliberate (5). As an outcome, discrimination has the potential to generate severe stress and can have a significant impact on health, particularly cardiovascular risk factors, including hypertension, obesity, and tissue inflammation (5).

A review by Paradise et al., investigated the association between self-reported racial discrimination and health and found that the association between discrimination and mental health was stronger than the association between discrimination and physical health. However, as pointed out by Williams et al., this review failed to include studies that measured discrimination, bias, and unfair treatment but did not explicitly identify race or ethnicity as causal. Importantly, many of the excluded studies were ones that used two important tools: (1) Everyday Discrimination Scale, and (2) Major Experiences of Discrimination Scale (70). When taken into account, it is increasingly clear that experiencing discrimination adversely impacts health, in particular cardiovascular disease risk factors, such as obesity, hypertension, smoking, alcohol abuse, and lack of sleep (70).

Over the past 10 years, the mechanism by which discrimination increase health disparities between Black/African Americans and Whites is becoming better understood (2, 4, 8). In a landmark paper, Krieger, proposed an “ecosocial theory of racism in which racism becomes embodied over the life course, adversely affecting the health of oppressed populations” (8). As a result, discrimination contributes to hypertension (9, 71–75), coronary artery disease (76–78), and metabolic abnormalities in low density lipoprotein cholesterol (9), increased abdominal adipose tissue deposition, insulin-resistance, and type-2 diabetes (5, 9, 79–81). Given these findings, discrimination is a known risk factor for poor cardiovascular outcomes (4, 82–84). Taken together, these studies highlight potential pathways through which discrimination may increase the incidence, severity, and mortality from cardiovascular disease.

Research on self-identified discrimination and health reveals that the threat imposed by discrimination can result in “vigilism,” depression, and chronic stress. All three of these states are linked with hypertension, loss of sleep, and obesity. Again, these clinical outcomes are risk factors for cardiovascular disease (5, 39). Further research in this area will be important going forward, particularly studies linking the release of stress hormones, disruption of circadian rhythms, and tissue inflammation with cardiovascular disease incidence and outcomes.

While many important studies link self-identified discrimination and cardiovascular risk factors with disease, there are limitations to the literature. Methods require self-reporting of many sensitive or private issues including life domains (e.g., healthcare, education, housing, and employment) and private domains (e.g., racial slurs and epithets, racial-profiling, or even physical violence) (5, 9). While important, these self-reporting measures are vulnerable to underreporting because not all individuals are willing to acknowledge these highly personal and potentially damaging events (5, 9). Furthermore, self-report may not adequately identify the full impact of discrimination (5, 9). The study by Lukachko et al., cited above, was important because it went beyond self-identified discrimination and systematically identified societal measures of discrimination (9). Importantly, the study also had representation across all 50 states and was sufficiently powered to account for potential confounders.

## Biological Mechanisms Linking Structural and Cultural Racism and Discrimination With Increased Cardiovascular Disease Incidence and Mortality

**FIGURE 3:**
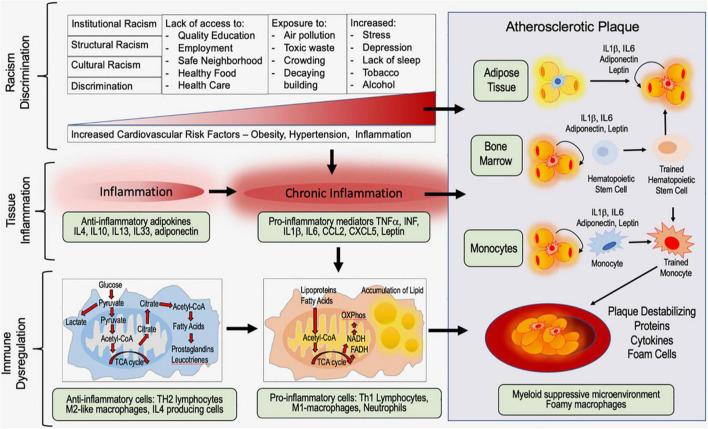
Working model of the impact of racism and discrimination on tissue inflammation, immune dysregulation, and atherosclerotic plaque formation.

Racism and self-reported discrimination are associated with increased risk for cardiovascular disease; biological mechanisms include telomere shortening, increased allostatic load, increased oxidative stress, and tissue inflammation (Figures 1–3). This is a rapidly evolving field; it is beyond the scope of this review to comprehensively discuss all the biological mechanisms linking racism and discrimination with cardiovascular disease. Instead, here we aim to focus on the impact of structural racism/discrimination on inflammation and the immune contribution to the biology of atherosclerotic plaque formation (Figures 2, 3). We focus on this biological mechanism because of its power to link racism/discrimination not only to cardiovascular disease but also to obesity and cancer (85, 86).

Traditional risk factors for cardiovascular disease include dyslipidemia, obesity, diabetes, hypertension, aging, and smoking, the majority of which are linked with racism and discrimination. Recently, there is increased appreciation of non-classical risk factors, such as infections and chronic inflammatory diseases (87), which opens up an important new area of investigation on potential biological mechanisms with which racism can further promote cardiovascular disease.

Atherosclerosis is a disease that involves components of the vascular, metabolic, and immune systems, and is characterized by the formation of atherosclerotic plaques in the arterial intima, eventually leading to heart failure, stroke and cardiac dysfunction. Atherosclerotic lesions comprise a necrotic core, the accumulation of modified lipoproteins and infiltration and adhesion of leukocytes including neutrophils and macrophages laden with cholesterol. Persistent low-grade inflammation is key in all stages of atherosclerotic plaque development, from initiation through progression and, ultimately, thrombosis.

The cardiovascular inflammatory process has been extensively reviewed by Soehnlein and Libby (88). As an overview, the early stages of atherosclerosis involve the secretion of chemokines by activated platelets and activated smooth muscle cells, such as C-C motif chemokine 5 (CCL5) that promote infiltration of monocytes and neutrophils. During the progression of the atherosclerotic lesion, smooth muscle cells undergo necrosis, increasing inflammation, and promote the formation of neutrophil extracellular traps (NETs), which play an important role in plaque formation and rupture.

Histones associated with NETs increase plaque formation by promoting homing monocytes, which differentiate into tissue-associate macrophages (TAM). TAMs actively uptake lipids and oxidized cholesterol-rich lipoproteins that adhere to arterial walls. Lipid-uptake by TAMs leads to the formation of foam cells; foam cells are polarized M1 inflammatory TAM that are filled with oxidized low-density lipoproteins (oxLDL).

Further, inflammatory cascades are directly activated by NETs. NETs activate the inflammasome complex (NACHT, LRR and PYD domains-containing protein 3 (NLRP3) and increase transcription of pro-interleukin-1 beta (IL-1β) by M1 macrophages. The activated NLRP3 inflammasome, in turn, activates caspase 1, which then cleaves pro-IL-1β into mature IL-1β. IL-1β is a cytokine that has a major role in the inflammatory milieu of atheroma. A recent clinical trial targeting IL-1β has shown efficacy against atherosclerosis (89), providing evidence that the inflammatory response plays a major role in the progression and outcome of cardiovascular disease. Taken together, inflammation is clearly a determinant of plaque instability – a thrombosis trigger – and ultimately, cardiovascular disease (88).

### Biological Link Between Discrimination, Racism, and Tissue Inflammation

Current conceptual models propose that perceived discrimination worsens health through both acute and chronic mechanisms that, in turn, impact multiple physiological and biological pathways (90). Acute events are typically described through a stress reactivity framework – an acute stressor leads to increased heart rate and blood pressure (91). Chronic stress is thought to increase vulnerability to disease by heightening both cardiac and inflammatory reactivity (92–95). An example of this model is the “weathering hypothesis” developed by Geronimus and colleagues; in this model they propose that the health inequity experienced by Black/African American individuals is a consequence of the “cumulative impact of social, economic, and political exclusion,” which leads to accelerated aging (96–99). Taken together, these models and frameworks provide mechanisms by which past and chronic discrimination may impact an individual’s biological response to acute new stressors (81, 100). Consistent with these models and frameworks, two meta-analyses show that perceived discrimination is significantly associated with dysregulation of the acute and chronic physiological stress systems (90).

Recent studies highlight the important relationship between discrimination, inflammation, and increased risk for cardiovascular disease. Cardiovascular risk is associated with elevated serum inflammatory proteins, including C-reactive protein (CRP), interleukin-6 (IL-6), IL-1β, and tumor necrosis factor-alpha (TNF-α). These circulating inflammatory markers are increased in Black/African American individuals relative to non-Hispanic Whites (101, 102). A recent study by Simons et al., investigated the link between systemic inflammation and discrimination in samples collected from Blacks/African Americans participating in the Family and Community Health Study (103). This study identified that juvenile exposure to discrimination and segregation was a strong predictor of adult chronic inflammation – including elevation of the inflammatory mediators CRP, IL-1β, IL-6, and TNF-α. Inflammation was more robustly predicted by discrimination and racial residential segregation than traditional cardiovascular risk factors including smoking, poor diet, lack of exercise, and low socioeconomic status.

A complete discussion of the impact of perceived discrimination on the brain-body pathways linking stress and physical health is beyond the scope of this review; for an excellent review on this topic please see Lockwood et al. (90).

### Trained Immunity, Plaque Formation, and Cardiovascular Disease

Recent studies of cardiovascular disease highlight the importance of tissue inflammation in “hyperactivation” of the immune system [for a review please see Bekkering et al. (104)]. Trained immunity – also called non-specific immunologic memory – is an emerging concept to explain the long-term adaptation of innate immune cells such as macrophages that leads to a sustained immune response (105). This sustained response is induced by metabolic programming and epigenetic changes evoked by chronic exposure. Studies, initially of bacterial infection, show that after an initial stimulus, a secondary stimulation occurs, which results in chronic sustained inflammation (106, 107).

During atherosclerosis, the endogenous stimulus, oxLDL, induces proatherogenic circulating monocytes (TAMs). These proatherogenic cells exhibit H3K4me3 methylation marks at the promoter regions of inflammatory genes, which open chromatin, increase production of inflammatory cytokines, and increase production of foam cells (108). Circulating monocytes from patients with severe atherosclerosis have been shown to exhibit trained immunity, including enhanced cytokine production capacity, glycolytic metabolism, and epigenetic reprogramming (109–111).

Normally, an increased and potent secondary response is beneficial against to infection. However, within a cardiac blood vessel of an at-risk individual, the sustained exposure of macrophages to chronic endogenous inflammatory stimuli (e.g., cytokines, chemokines, oxLDL, cholesterol crystals, and tissue damage-derived signals) has been recently linked to non-resolving vascular inflammation and atherosclerosis (104). Importantly, early life experiences and exposures are linked to persistent immune activation and trained immunity (104, 112). These processes are now being recognized as key contributors to cardiovascular disease risk, atherosclerotic plaque formation, and, ultimately, plaque rupture.

## Discussion

Over the past 20 years, the overall death rate from cardiovascular disease has declined. These health gains, however, have not been equally shared by Black/African Americans. Black/African American communities experience a 30% higher death rate from cardiovascular disease versus non-Hispanic Whites – even when controlling for socioeconomic status (3). The recent COVID-19 pandemic has once again highlighted the impact of structural racism and racial residential segregation in promoting health disparities and unequal deaths (113).

The deterioration of Black/African American neighborhoods did not happen overnight and are the result of longstanding racist and discriminatory policies implemented at the national, state, and individual level, such as red lining and chronic disinvestment. As stated by Keith Churchwell, M.D., FAHA, Chair of the AHA Advisory Committee, “structural racism is an embedded part of legal, business, and social constructs and a feature of the social, economic, and political systems in which we all exist.” As a result, there is a direct connection between disparities in housing and disparities in cardiovascular disease incidence and mortality. To remedy these inequities, formerly redlined neighborhoods need to be made safe and walkable. Highways that run through urban spaces need to be rerouted and neighborhoods reconnected (114). Parks and green spaces need to be located in formerly redlined neighborhoods to reduce the impact of heat waves and provide a safe space for exercise. To make neighborhoods truly safe for exercise, cardiotoxins in the air also need to be eliminated; to accomplish this we need to reinstitute clean air standards, incentivize low emission cars, and increase clean emission standards for diesel trucks. There also needs to be legislation to rezone residential formerly redlined neighborhoods to prevent factories and power plants from locating near homes. As factories close, we should replace the building’s occupant with clean industry or repurpose the building to house small businesses, apartments, and grocery stores.

The work of Stokes et al. provides evidence that improvements in cardiovascular health are possible through crowdsourced health care facility ratings (115). In a recent study, these investigators showed that online ratings and review may provide important insight into unequal health care. This study provides a powerful strategy by which citizens can help to end unequal and discriminatory care (115).

Fundamental to eliminating structural racism – while ambitious but necessary – is the ability to vote. Unfortunately, we’re watching voting rights come, once again, under fire in the United States. It is critical that voting rights remain intact so that Black/African Americans and other racial/ethnic minorities can participate in democracy and vote in legislatures and ballot resolutions that act to eliminate structural racism and health disparities.

Capacity-building is also vitally important for reducing disparities in cardiac disease. Medicaid expansion (or “Obamacare”) needs to be implemented in all 50 states. The training of Black/African American primary care physicians, internists, and cardiologists needs to be prioritized. The pipeline needs to start early – through mentored science, technology, engineering, and math (STEM) pipeline programs. High school and college students need to have access to programs that teach and inspire. An example of such a program is the MED program at University of North Carolina at Chapel Hill (UNC-MED) (116).

The biological mechanisms underlying the impact of housing policies on tissue inflammation, immune suppression, and creation of atherosclerotic plaques have only just begun. Whether Black/African American individuals are prone to trained immunity – explaining in part, increased atherosclerosis in Black/African Americans vs. non-Hispanic Whites – has not yet been studied. The field of trained immunity and cardiovascular disease is still rapidly evolving. However, given the impact of chronic discrimination on inflammation and elevated production of chronic elevated production of inflammatory factors and cytokines, it is easy to connect the dots between chronic discrimination, trained immunity, and disparities in cardiovascular disease. Future studies, hopefully by the next generation of Black/African American scientists, will take the lead in uncovering these biological mechanisms.

## Author Contributions

VS, CS, and NT wrote much of the manuscript together. RW contributed key ideas, oversaw the writing of the manuscript, and edited the manuscript. TH, LE, VJ, GM-C, EM, ED, SG, and NW contributed ideas and edited and revised the manuscript. AO and MS-P wrote and edited the section on inflammation and trained immunity in the revision. All authors approved the addition of AO, MS-P, EM, SG, and ED. All authors contributed to the article and approved the submitted version.

## Conflict of Interest

The authors declare that the research was conducted in the absence of any commercial or financial relationships that could be construed as a potential conflict of interest.

## Publisher’s Note

All claims expressed in this article are solely those of the authors and do not necessarily represent those of their affiliated organizations, or those of the publisher, the editors and the reviewers. Any product that may be evaluated in this article, or claim that may be made by its manufacturer, is not guaranteed or endorsed by the publisher.

## References

[1] Lawrence K Keleher T. *Structural Racism.* (2004). Available online at: http://www.intergroupresources.com/rc/Definitions%20of%20Racism.pdf. (accessed April 8, 2021)

[2] Jones CP. Levels of racism: a theoretic framework and a gardener’s tale. *Am J Public Health.* (2000) 90:1212–5. 10.2105/ajph.90.8.1212 10936998 PMC1446334

[3] Churchwell K Elkind MSV Benjamin RM Carson AP Chang EK Lawrence W Call to action: structural racism as a fundamental driver of health disparities: a presidential advisory from the American heart association. *Circulation.* (2020) 142:e454–68. 10.1161/CIR.0000000000000936 33170755

[4] Williams DR Mohammed SA. Discrimination and racial disparities in health: evidence and needed research. *J Behav Med.* (2009) 32:20–47. 10.1007/s10865-008-9185-0 19030981 PMC2821669

[5] Williams DR Lawrence JA Davis BA. Racism and health: evidence and needed research. *Annu Rev Public Health.* (2019) 40:105–25. 10.1146/annurev-publhealth-040218-043750 30601726 PMC6532402

[6] Arias E Xu J Jim MA. Period life tables for the non-Hispanic American Indian and Alaska native population, 2007-2009. *Am J Public Health.* (2014) 104(Suppl. 3):S312–9. 10.2105/AJPH.2013.301635 24754553 PMC4035861

[7] Braveman PA Cubbin C Egerter S Williams DR Pamuk E. Socioeconomic disparities in health in the United States: what the patterns tell us. *Am J Public Health.* (2010) 100(Suppl. 1):S186–96. 10.2105/AJPH.2009.166082 20147693 PMC2837459

[8] Krieger N. Methods for the scientific study of discrimination and health: an ecosocial approach. *Am J Public Health.* (2012) 102:936–44. 10.2105/AJPH.2011.300544 22420803 PMC3484783

[9] Lukachko A Hatzenbuehler ML Keyes KM. Structural racism and myocardial infarction in the United States. *Soc Sci Med.* (2014) 103:42–50. 10.1016/j.socscimed.2013.07.021 24507909 PMC4133127

[10] Kramer MR Black NC Matthews SA James SA. The legacy of slavery and contemporary declines in heart disease mortality in the U.S. south. *SSM Popul Health.* (2017) 3:609–17. 10.1016/j.ssmph.2017.07.004 29226214 PMC5718368

[11] Vaughan AS Quick H Pathak EB Kramer MR Casper M. Disparities in temporal and geographic patterns of declining heart disease mortality by race and sex in the United States, 1973-2010. *J Am Heart Assoc.* (2015) 4:e002567. 10.1161/JAHA.115.002567 26672077 PMC4845281

[12] Center for Disease Control and Prevention. *Health, United States Spotlight: Racial and Ethnic Disparities in Heart Disease.* (2019). Available online at: https://www.cdc.gov/nchs/hus/spotlight/HeartDiseaseSpotlight_2019_0404.pdf (accessed April 8, 2021)

[13] History.com Editors. *Fugitive Slave Acts.* (2020). Available online at: https://www.history.com/topics/black-history/fugitive-slave-acts (accessed March 12, 2021)

[14] Tischauser LV. *Jim Crow Laws.* Santa Barbara, CA: Greenwood (2012).

[15] Williams DR. Race and health: basic questions, emerging directions. *Ann Epidemiol.* (1997) 7:322–33. 10.1016/s1047-2797(97)00051-3 9250627

[16] Williams DR. Miles to go before we sleep: racial inequities in health. *J Health Soc Behav.* (2012) 53:279–95. 10.1177/0022146512455804 22940811 PMC3712789

[17] Jackson KT. *Crabgrass Frontier: The Suburbanization of the United States.* New York, NY: Oxford University Press (1985). 352 p.

[18] Hiller AE. *Redlining and the Homeowners’ Loan Corporation.* (2003). Available online at: https://repository.upenn.edu/cgi/viewcontent.cgi?article=1002&context=cplan_papers (accessed April 8, 2021).

[19] Crossney KB Bartelt DW. Residential security, risk, and race: the home owners’ loan corporation and mortgage access in two cities. *Urban Geogr.* (2005) 26:707–36. 10.2747/0272-3638.26.8.707

[20] Crossney KB Bartelt DW. The legacy of the home owners’ loan corporation. *Housing Policy Debate.* (2005) 16:547–74. 10.1080/10511482.2005.9521555

[21] Schill MH Wachter SM. Principles to guide housing policy at the beginning of the millennium. *Cityscape.* (2001) 5:5–19.

[22] Plotkin W. *Racial” Provisions of FHA Underwriting Manual, 1938.* (2003). Available online at: http://wbhsi.net/~wendyplotkin/DeedsWeb/fha38.html (accessed April 8, 2021)

[23] Hanlon B Short JR. *Housing Policy and the Suburban Metropolis: A Focus on the United States and France. The Routledge Handbook of Housing Policy and Planning.* Milton Park: Routledge (2019). p. 347–56.

[24] Hanchett TW. The other ‘Subsidized Housing’: federal aid to suburbanization, 1940s-1960s. In: Bauman J, Biles R, Szlvian K editors. *From Tenements to the Taylor Homes: in Search of an Urban Housing Policy in Twentieth Century America.* University Park, PA: Pennsylvania State University Press (2000). p. 163–79.

[25] Artibise AFJ. Gelfand, mark. a Nation of cities: the federal government and Urban America, 1933-1965. *Urban Hist Rev.* (1976) 5:30–1. 10.7202/1019551ar 33270396

[26] Rothstein R. *The Color Of Law: A Forgotten History Of How Our Government Segregated America.* New York, NY: Liveright Publishing (2017).

[27] Hanks A Solomon D Weller CE. *Systematic Inequality: How America’s Structural Racism Helped Create the Black-White Wealth Gap.* (2018). Available online at: https://www.americanprogress.org/issues/race/reports/2018/02/21/447051/systematic-inequality/ (accessed May 8, 2021)

[28] Badger E. *Whites Have Huge Wealth Edge Over Blacks (But Don’t Know It).* (2017). Available online at: https://www.nytimes.com/interactive/2017/09/18/upshot/black-white-wealth-gap-perceptions.html (accessed May 8, 2021)

[29] Archer DN. White men’s roads through black men’s homes”: advancing racial equity through highway reconstruction. *Vand L Rev.* (2020) 73:1259.

[30] Jaffe H. *The Insane Highway Plan That Would Have Bulldozed DC’s Most Charming Neighborhoods.* (2015). Available online at: https://www.washingtonian.com/2015/10/21/the-insane-highway-plan-that-would-have-bulldozed-washington-dcs-most-charming-neighborhoods/ (accessed May 8, 2021)

[31] Senate Historical Office. *Congress Approves the Federal-Aid Highway Act.* (1956). Available online at: https://www.senate.gov/artandhistory/history/minute/Federal_Highway_Act.htm (accessed May 8, 2021)

[32] Tessum CW Paolella DA Chambliss SE Apte JS Hill JD Marshall JD. PM2.5 polluters disproportionately and systemically affect people of color in the United States. *Sci Adv.* (2021) 7:eabf4491. 10.1126/sciadv.abf4491 33910895 PMC11426197

[33] Carnethon MR Pu J Howard G Albert MA Anderson CAM Bertoni AG Cardiovascular health in African Americans: a scientific statement from the American heart association. *Circulation.* (2017) 136:e393–423. 10.1161/CIR.0000000000000534 29061565

[34] Primary Care Development Corporation. *Today’s Health Inequities in New York City Driven by Historic Redlining Practices.* (2020). Available online at: https://www.pcdc.org/wp-content/uploads/Points-on-Care-_-Issue-5-_-FINAL.pdf (accessed June 8, 2021)

[35] Mujahid MS Gao X Tabb LP Morris C Lewis TT. Historical redlining and cardiovascular health: the multi-ethnic study of atherosclerosis. *Proc Natl Acad Sci USA.* (2021) 118:e2110986118. 10.1073/pnas.2110986118 34903653 PMC8713797

[36] Hoffman JS Shandas V Pendleton N. The effects of historical housing policies on resident exposure to intra-urban heat: a study of 108 US urban areas. *Climate.* (2020) 8:12. 10.3390/cli8010012

[37] Firebaugh G Acciai F. For blacks in America, the gap in neighborhood poverty has declined faster than segregation. *Proc Natl Acad Sci USA.* (2016) 113:13372–7. 10.1073/pnas.1607220113 27821759 PMC5127296

[38] Bishaw A. *Changes in Areas with Concentrated Poverty: 2000 to 2010.* (2014). Available online at: https://www2.census.gov/library/publications/2014/acs/acs-27.pdf (accessed June 3, 2022).

[39] Lawrence K Keleher T. *Structural Racism.* (2004). Available online at: https://static1.squarespace.com/static/585a5248579fb3abe813c2c4/t/5ed478acdf699b54f8d25430/1590982828921/Definitions-of+Racism.pdf (accessed March 22, 2022).

[40] James CE. *Perspectives on Racism and the Human Services Sector: A Case for Change.* Toronto, ON: University of Toronto Press (1996).

[41] Massey DS Denton NA. The dimensions of residential segregation. *Soc Forces.* (1988) 67:281–315. 10.2307/2579183

[42] Massey DS Denton NA. Hypersegregation in U.S. metropolitan areas: black and hispanic segregation along five dimensions. *Demography.* (1989) 26:373–91. 10.2307/2061599 2792476

[43] Massey D Denton NA. *American apartheid: Segregation and the Making of the Underclass.* Cambridge, MA: Harvard University Press (1993).

[44] White K Borrell LN. Racial/ethnic residential segregation: framing the context of health risk and health disparities. *Health Place.* (2011) 17:438–48. 10.1016/j.healthplace.2010.12.002 21236721 PMC3056936

[45] Harrell CJP Burford TI Cage BN Nelson TM Shearon S Thompson A Multiple pathways linking racism to health outcomes. *Du Bois Rev.* (2011) 8:143–57. 10.1017/S1742058X11000178 22518195 PMC3328094

[46] Orfield G Frankenberg E Garces LM. Statement of American social scientists of research on school desegregation to the US Supreme Court in Parents v. seattle school district and meredith v. *Jefferson County Urban Rev.* (2008) 40:96–136. 10.1007/s11256-007-0073-7

[47] White K Haas JS Williams DR. Elucidating the role of place in health care disparities: the example of racial/ethnic residential segregation. *Health Serv Res.* (2012) 47(3 Pt 2):1278–99. 10.1111/j.1475-6773.2012.01410.x 22515933 PMC3417310

[48] Lewis TT Williams DR Tamene M Clark CR. Self-reported experiences of discrimination and cardiovascular disease. *Curr Cardiovasc Risk Rep.* (2014) 8:365. 10.1007/s12170-013-0365-2 24729825 PMC3980947

[49] Link BG Phelan J. Social conditions as fundamental causes of disease. *J Health Soc Behav.* (1995):Spec No:80–94. 7560851

[50] Heron MP. Deaths: leading causes for 2017. *Natl Vital Stat Rep.* (2019) 68:1–77. 32501203

[51] Kershaw KN Osypuk TL Do DP De Chavez PJ Diez Roux AV. Neighborhood-level racial/ethnic residential segregation and incident cardiovascular disease: the multi-ethnic study of atherosclerosis. *Circulation.* (2015) 131:141–8. 10.1161/CIRCULATIONAHA.114.011345 25447044 PMC4293329

[52] Kershaw KN Albrecht SS. Racial/ethnic residential segregation and cardiovascular disease risk. *Curr Cardiovasc Risk Rep.* (2015) 9:10. 10.1007/s12170-015-0436-7 25893031 PMC4399822

[53] Mayne SL Hicken MT Merkin SS Seeman TE Kershaw KN Do DP Neighbourhood racial/ethnic residential segregation and cardiometabolic risk: the multiethnic study of atherosclerosis. *J Epidemiol Community Health.* (2019) 73:26–33. 10.1136/jech-2018-211159 30269056 PMC6398328

[54] Baxter SLK Chung R Frerichs L Thorpe RJ Jr. Skinner AC Weinberger M. Racial residential segregation and race differences in ideal cardiovascular health among young men. *Int J Environ Res Public Health.* (2021) 18:7755. 10.3390/ijerph18157755 34360047 PMC8345482

[55] Rosamond WD Chambless LE Heiss G Mosley TH Coresh J Whitsel E Twenty-two-year trends in incidence of myocardial infarction, coronary heart disease mortality, and case fatality in 4 US communities, 1987-2008. *Circulation.* (2012) 125:1848–57. 10.1161/CIRCULATIONAHA.111.047480 22420957 PMC3341729

[56] Thorpe RJ Jr. Kelley E Bowie JV Griffith DM Bruce M LaVeist T. Explaining racial disparities in obesity among men: does place matter? *Am J Mens Health.* (2015) 9:464–72. 10.1177/1557988314551197 25249452 PMC4864070

[57] Thorpe RJ Jr. Kennedy-Hendricks A Griffith DM Bruce MA Coa K Bell CN Race, Social and Environmental Conditions, and Health Behaviors in Men. *Fam Community Health.* (2015) 38:297–306. 10.1097/FCH.0000000000000078 26291190 PMC5052072

[58] Gee GC Walsemann KM Brondolo E. A life course perspective on how racism may be related to health inequities. *Am J Public Health.* (2012) 102:967–74. 10.2105/AJPH.2012.300666 22420802 PMC3483932

[59] Williams DR Mohammed SA. Racism and health I: pathways and scientific evidence. *Am Behav Sci.* (2013) 57:1152–73. 10.1177/0002764213487340 24347666 PMC3863357

[60] Hicken MT Kravitz-Wirtz N Durkee M Jackson JS. Racial inequalities in health: framing future research. *Soc Sci Med.* (2018) 199:11–8. 10.1016/j.socscimed.2017.12.027 29325781 PMC5915332

[61] Pyke KD. What is internalized racial oppression and why don’t we study it? Acknowledging racism’s hidden injuries. *Sociol Persp.* (2010) 53:551–72. 10.1525/sop.2010.53.4.551

[62] Speight SL. Internalized racism: one more piece of the puzzle. *Counseling Psychol.* (2007) 35:126–34. 10.1177/0011000006295119

[63] Krysan M Farley R Couper MP. In the eye of the beholder: racial beliefs and residential segregation. *Du Bois Rev.* (2008) 5:5–26. 10.1017/s1742058x08080028

[64] Williams DR Wyatt R. Racial bias in health care and health: challenges and opportunities. *JAMA.* (2015) 314:555–6. 10.1001/jama.2015.9260 26262792

[65] Smedley B Stith A Nelson A. *Unequal Treatment: Confronting Racial and Ethnic Disparities in Healthcare.* Washington, DC: National Academies Press (2003). 25032386

[66] Van Ryn M Burgess DJ Dovidio JF Phelan SM Saha S Malat J The impact of racism on clinician cognition, behavior, and clinical decision making. *Du Bois Rev.* (2011) 8:199–218. 10.1017/S1742058X11000191 24761152 PMC3993983

[67] Cooper LA Roter DL Carson KA Beach MC Sabin JA Greenwald AG The associations of clinicians’ implicit attitudes about race with medical visit communication and patient ratings of interpersonal care. *Am J Public Health.* (2012) 102:979–87. 10.2105/AJPH.2011.300558 22420787 PMC3483913

[68] Egede LE Walker RJ Williams JS. Intersection of structural racism, social determinants of health, and implicit bias with emergency physician admission tendencies. *JAMA Netw Open.* (2021) 4:e2126375. 10.1001/jamanetworkopen.2021.26375 34546376 PMC8594614

[69] Mandavilli A. *Editor of JAMA Leaves After Outcry Over Colleague’s Remarks on Racism: The New York Times.* (2021). Available online at: https://www.nytimes.com/2021/06/01/health/jama-bauchner-racism.html (accessed November 18, 2021)

[70] Williams DR Yan Y Jackson JS Anderson NB. Racial differences in physical and mental health: socio-economic status, stress and discrimination. *J Health Psychol.* (1997) 2:335–51. 10.1177/135910539700200305 22013026

[71] Davis SK Liu Y Quarells RC Din-Dzietham R. Stress-related racial discrimination and hypertension likelihood in a population-based sample of African Americans: the metro atlanta heart disease study. *Ethn Dis.* (2005) 15:585–93. 16259480

[72] Guyll M Matthews KA Bromberger JT. Discrimination and unfair treatment: relationship to cardiovascular reactivity among African American and European American women. *Health Psychol.* (2001) 20:315. 10.1037//0278-6133.20.5.315 11570645

[73] Krieger N. Racial and gender discrimination: risk factors for high blood pressure? *Soc Sci Med.* (1990) 30:1273–81. 10.1016/0277-9536(90)90307-e 2367873

[74] Krieger N Sidney S. Racial discrimination and blood pressure: the CARDIA study of young black and white adults. *Am J Public Health.* (1996) 86:1370–8. 10.2105/ajph.86.10.1370 8876504 PMC1380646

[75] Roberts CB Vines AI Kaufman JS James SA. Cross-sectional association between perceived discrimination and hypertension in African-American men and women: the pitt county study. *Am J Epidemiol.* (2008) 167:624–32. 10.1093/aje/kwm334 18083714

[76] Troxel WM Matthews KA Bromberger JT Sutton-Tyrrell K. Chronic stress burden, discrimination, and subclinical carotid artery disease in African American and caucasian women. *Health Psychol.* (2003) 22:300. 10.1037/0278-6133.22.3.300 12790258

[77] Ayotte BJ Hausmann LR Whittle J Kressin NR. The relationship between perceived discrimination and coronary artery obstruction. *Am Heart J.* (2012) 163:677–83. 10.1016/j.ahj.2012.01.006 22520534

[78] Lewis TT Everson-Rose SA Powell LH Matthews KA Brown C Karavolos K Chronic exposure to everyday discrimination and coronary artery calcification in African-American women: the SWAN Heart Study. *Psychosomatic medicine* (2006) 68:362–8. 10.1097/01.psy.0000221360.94700.16 16738065

[79] Lewis TT Aiello AE Leurgans S Kelly J Barnes LL. Self-reported experiences of everyday discrimination are associated with elevated C-reactive protein levels in older African-American adults. *Brain Behav Immun.* (2010) 24:438–43. 10.1016/j.bbi.2009.11.011 19944144 PMC2826562

[80] Lewis TT Kravitz HM Janssen I Powell LH. Self-reported experiences of discrimination and visceral fat in middle-aged African-American and caucasian women. *Am J Epidemiol.* (2011) 173:1223–31. 10.1093/aje/kwq466 21354991 PMC3101065

[81] Richman LS Bennett GG Pek J Siegler I Williams RB. Discrimination, dispositions, and cardiovascular responses to stress. *Health Psychol.* (2007) 26:675–83. 10.1037/0278-6133.26.6.675 18020838

[82] Sawyer PJ Major B Casad BJ Townsend SS Mendes WB. Discrimination and the stress response: Psychological and physiological consequences of anticipating prejudice in interethnic interactions. *Am J Public Health.* (2012) 102:1020–6. 10.2105/AJPH.2011.300620 22420818 PMC3483920

[83] Dimsdale JE. Psychological stress and cardiovascular disease. *J Am Coll Cardiol.* (2008) 51:1237–46. 18371552 10.1016/j.jacc.2007.12.024 PMC2633295

[84] Calvin R Winters K Wyatt SB Williams DR Henderson FC Walker ER. Racism and cardiovascular disease in African Americans. *Am J Med Sci.* (2003) 325:315–31. 10.1097/00000441-200306000-00003 12811228

[85] Dietze EC Chavez TA Seewaldt VL. Obesity and triple-negative breast cancer: disparities, controversies, and biology. *Am J Pathol.* (2018) 188:280–90. 10.1016/j.ajpath.2017.09.018 29128565 PMC5785535

[86] Zavala VA Bracci PM Carethers JM Carvajal-Carmona L Coggins NB Cruz-Correa MR Cancer health disparities in racial/ethnic minorities in the United States. *Br J Cancer.* (2021) 124:315–32. 10.1038/s41416-020-01038-6 32901135 PMC7852513

[87] Zhong C Yang X Feng Y Yu J. Trained immunity: an underlying driver of inflammatory atherosclerosis. *Front Immunol.* (2020) 11:284. 10.3389/fimmu.2020.00284 32153588 PMC7046758

[88] Soehnlein O Libby P. Targeting inflammation in atherosclerosis - from experimental insights to the clinic. *Nat Rev Drug Discov.* (2021) 20:589–610. 10.1038/s41573-021-00198-1 33976384 PMC8112476

[89] Libby P. Inflammation in Atherosclerosis-No Longer a Theory. *Clin Chem* (2021) 67:131–42. 10.1093/clinchem/hvaa275 33393629

[90] Lockwood KG Marsland AL Matthews KA Gianaros PJ. Perceived discrimination and cardiovascular health disparities: a multisystem review and health neuroscience perspective. *Ann N Y Acad Sci.* (2018) 1428:170–207. 10.1111/nyas.13939 30088665

[91] Pascoe EA Smart Richman L. Perceived discrimination and health: a meta-analytic review. *Psychol Bull.* (2009) 135:531–54. 10.1037/a0016059 19586161 PMC2747726

[92] Cohen S Hamrick N. Stable individual differences in physiological response to stressors: implications for stress-elicited changes in immune related health. *Brain Behav Immun.* (2003) 17:407–14. 10.1016/s0889-1591(03)00110-7 14583231

[93] Herbert TB Cohen S Marsland AL Bachen EA Rabin BS Muldoon MF Cardiovascular reactivity and the course of immune response to an acute psychological stressor. *Psychosom Med.* (1994) 56:337–44. 10.1097/00006842-199407000-00009 7972616

[94] Cohen S Hamrick N Rodriguez MS Feldman PJ Rabin BS Manuck SB. The stability of and intercorrelations among cardiovascular, immune, endocrine, and psychological reactivity. *Ann Behav Med.* (2000) 22:171–9. 10.1007/BF02895111 11211850

[95] Uchino BN Cacioppo JT Malarkey W Glaser R. Individual differences in cardiac sympathetic control predict endocrine and immune responses to acute psychological stress. *J Pers Soc Psychol.* (1995) 69:736–43. 10.1037//0022-3514.69.4.736 7473028

[96] Geronimus AT Hicken M Keene D Bound J. “Weathering” and age patterns of allostatic load scores among blacks and whites in the United States. *Am J Public Health.* (2006) 96:826–33. 10.2105/AJPH.2004.060749 16380565 PMC1470581

[97] Geronimus AT Hicken MT Pearson JA Seashols SJ Brown KL Cruz TD. Do US black women experience stress-related accelerated biological aging: A novel theory and first population-based test of black-white differences in telomere length. *Hum Nat.* (2010) 21:19–38. 10.1007/s12110-010-9078-0 20436780 PMC2861506

[98] Geronimus AT James SA Destin M Graham LA Hatzenbuehler M Murphy M Jedi public health: Co-creating an identity-safe culture to promote health equity. *SSM Popul Health.* (2016) 2:105–16. 10.1016/j.ssmph.2016.02.008 27022616 PMC4807633

[99] Geronimus AT Thompson JP. To denigrate, ignore, or disrupt: racial inequality in health and the impact of a policy-induced breakdown of African American communities. *Du Bois Rev.* (2004) 1:247–79.

[100] Lepore SJ Revenson TA Weinberger SL Weston P Frisina PG Robertson R Effects of social stressors on cardiovascular reactivity in black and white women. *Ann Behav Med.* (2006) 31:120–7. 10.1207/s15324796abm3102_3 16542126 PMC2593111

[101] Chyu L Upchurch DM. Racial and ethnic patterns of allostatic load among adult women in the United States: findings from the national health and nutrition examination Survey 1999-2004. *J Womens Health (Larchmt).* (2011) 20:575–83. 10.1089/jwh.2010.2170 21428732 PMC3075046

[102] Paalani M Lee JW Haddad E Tonstad S. Determinants of inflammatory markers in a bi-ethnic population. *Ethn Dis.* (2011) 21:142–9. 21749016 PMC3427005

[103] Simons RL Lei MK Beach SRH Barr AB Simons LG Gibbons FX Discrimination, segregation, and chronic inflammation: testing the weathering explanation for the poor health of black Americans. *Dev Psychol.* (2018) 54:1993–2006. 10.1037/dev0000511 30234347 PMC7685230

[104] Bekkering S Saner C Riksen NP Netea MG Sabin MA Saffery R Trained immunity: linking obesity and cardiovascular disease across the life-Course? *Trends Endocrinol Metab.* (2020) 31:378–89. 10.1016/j.tem.2020.01.008 32305098

[105] Netea MG Quintin J van der Meer JW. Trained immunity: a memory for innate host defense. *Cell Host Microbe.* (2011) 9:355–61. 10.1016/j.chom.2011.04.006 21575907

[106] Quintin J Saeed S Martens JHA Giamarellos-Bourboulis EJ Ifrim DC Logie C Candida albicans infection affords protection against reinfection via functional reprogramming of monocytes. *Cell Host Microbe.* (2012) 12:223–32. 10.1016/j.chom.2012.06.006 22901542 PMC3864037

[107] Ifrim DC Quintin J Joosten LA Jacobs C Jansen T Jacobs L Trained immunity or tolerance: opposing functional programs induced in human monocytes after engagement of various pattern recognition receptors. *Clin Vaccine Immunol.* (2014) 21:534–45. 10.1128/CVI.00688-13 24521784 PMC3993125

[108] Bekkering S Quintin J Joosten LA van der Meer JW Netea MG Riksen NP. Oxidized low-density lipoprotein induces long-term proinflammatory cytokine production and foam cell formation via epigenetic reprogramming of monocytes. *Arterioscler Thromb Vasc Biol.* (2014) 34:1731–8. 10.1161/ATVBAHA.114.303887 24903093

[109] Bekkering S van den Munckhof I Nielen T Lamfers E Dinarello C Rutten J Innate immune cell activation and epigenetic remodeling in symptomatic and asymptomatic atherosclerosis in humans in vivo. *Atherosclerosis.* (2016) 254:228–36. 27764724 10.1016/j.atherosclerosis.2016.10.019

[110] Shirai T Nazarewicz RR Wallis BB Yanes RE Watanabe R Hilhorst M The glycolytic enzyme PKM2 bridges metabolic and inflammatory dysfunction in coronary artery disease. *J Exp Med.* (2016) 213:337–54. 10.1084/jem.20150900 26926996 PMC4813677

[111] Davis FM Gallagher KA. Epigenetic Mechanisms in monocytes/macrophages regulate inflammation in cardiometabolic and vascular disease. *Arterioscler Thromb Vasc Biol.* (2019) 39:623–34. 10.1161/ATVBAHA.118.312135 30760015 PMC6438376

[112] Claycombe K King LE Fraker PJ. A role for leptin in sustaining lymphopoiesis and myelopoiesis. *Proc Natl Acad Sci USA.* (2008) 105:2017–21. 10.1073/pnas.0712053105 18250302 PMC2538874

[113] Newman LA Winn RA Carethers JM. Similarities in Risk for COVID-19 and cancer disparities. *Clin Cancer Res.* (2021) 27:24–7. 10.1158/1078-0432.CCR-20-3421 33051304 PMC7785577

[114] Brand M. *Highways Tore Through America’s Black Neighborhoods. Biden’s Infrastructure Plan Aims to Address That Inequity.* (2021). Available online at: https://www.kcrw.com/news/shows/press-play-with-madeleine-brand/biden-infrastructure-major-league-baseball/black-neighborhoods-gentrification-displacement-highways (accessed August 8, 2021)

[115] Stokes DC Pelullo AP Mitra N Meisel ZF South EC Asch DA Association between crowdsourced health care facility ratings and mortality in US Counties. *JAMA Netw Open.* (2021) 4:e2127799. 10.1001/jamanetworkopen.2021.27799 34665240 PMC8527362

[116] Office of Scholastic Enrichment and Equity, UNC School of Medicine. *Medical Education Development (MED) Program.* (2021). Available online at: https://www.med.unc.edu/inclusion/programs-initiatives/medical-education-development-program/ (accessed August 8, 2021)

